# Crosstalk between Edc4 and Mammalian Target of Rapamycin Complex 1 (mTORC1) Signaling in mRNA Decapping

**DOI:** 10.3390/ijms151223179

**Published:** 2014-12-12

**Authors:** Hazir Rahman, Muhammad Qasim, Michael Oellerich, Abdul R. Asif

**Affiliations:** 1 Institute for Clinical Chemistry/UMG-Laboratories, University Medical Center, Robert-Koch-Str. 40, Goettingen 37075, Germany; E-Mails: hazir.rahman@med.uni-goettingen.de (H.R.); muhammad.qasim@med.uni-goettingen.de (M.Q.); michael.oellerich@med.uni-goettingen.de (M.O.); 2 Department of Microbiology, Kohat University of Science and Technology, Kohat 26000, Pakistan

**Keywords:** mammalian target of rapamycin complex 1 (mTORC1) purification, mTORC1 interacting proteins, enhancer of mRNA decapping protein 4 (Edc4), mRNA decapping

## Abstract

The mammalian target of rapamycin complex 1 (mTORC1) is involved in the cellular transcription and translation processes. The undertaken study characterized the enhancer of mRNA decapping protein 4 (Edc4) as mTORC1 interacting protein. Human T lymphoblast (CCRF-CEM) cells were used for mTORC1 purification. Co-immunoprecipitation coupled with immunoblotting analysis was used to confirm the interaction of Edc4 in mTORC1 specific purifications. Further assays were incorporated to conclude the role of mTORC1 in mRNA decapping via Edc4. Edc4 was identified as a new interacting protein with mTORC1 in both the endogenous and myc-tag raptor component mTORC1 specific purifications. Quantitative co-localization using confocal microscopy demonstrated that raptor component of mTORC1 coexists with Edc4 in processing (P) bodies, a site for mRNA degradation. Incubation of cells with rapamycin, a known inhibitor of mTOR kinase activity, increased the total Edc4 protein expression but at the same time decreased the Edc4 interaction with mTORC1. Moreover, rapamycin treatment resulted in a significant decrease in total serine phosphorylated Edc4 protein signal and the total 5'-capped mRNA. These findings provide the first evidence for the pivotal role of mTORC1 in Edc4 regulation. Further in-depth studies are required to get a complete understanding of molecular crosstalk between mTORC1 signaling and mRNA decapping pathway.

## 1. Introduction

The mammalian target of rapamycin (mTOR) is a protein kinase regulates cell growth and cell survival [1,2]. mTOR is the catalytic subunit of two distinct protein complexes, mTOR complex 1 (mTORC1), and mTOR complex 2 (mTORC2) [3,4]. The mutually exclusive accessory proteins including regulatory associated proteins of mTOR (raptor) and rapamycin insensitive companion of mTOR (rictor) define mTORC1 and mTORC2, respectively [3,4]. mTORC1 is a rapamycin sensitive complex involved in the process of nutrient sensing, energy synthesis, translation, transcription, and lipid biosynthesis [5,6]. The expanding role of mTORC1 in several biological processes emphasizing the importance of identifying and understanding the function of the protein components of the mTORC1 signaling pathway.

Capping [5' N^7^-methyl-guanosine (m^7^GpppN)] of mRNA is required for the integrity of newly synthesized mRNA [7]. This protects mRNA from exonucleolytic degradation and promotes the translation of most cellular mRNAs [8]. mTORC1 is involved in the cap dependent translation of mRNA and regulates the eIF4E-binding protein 1 (4E-BP1), an inhibitor of eukaryotic initiation factor 4E (elF4E) [9]. eIF4E is a member of the translation initiation complex that protects the 5' mRNA cap from decapping and recruits the translation machinery necessary for efficient translation initiation [10]. mRNA decapping provides crucial control of mRNA turnover as decapping irreversibly removes the cap and promotes mRNA decay [8,11]. Decapping of mRNA is considered to take place in the processing bodies (P bodies) which are the cytosolic self-assembled aggregations of messenger ribonuclear proteins (mRNPs) involved in mRNA turnover, RNA interference (RNAi), micro RNA (miRNA) mediated gene silencing, and translation repression [8,11,12,13].

In higher eukaryotes, decapping requires enhancer of mRNA decapping protein 4 (Edc4) for catalytic complex formation between Dcp1a and Dcp2, which are two major proteins of the mRNA decapping complex [14]. Edc4 was originally identified as the autoantigen in the sera of Sjogren’s syndrome (a chronic autoimmune disease) patients [15]. Recently, Edc4 was suggested to be involved in miRNA-mediated translation repression [16]. Edc4 is essential for the integrity of P bodies [17]. The localization of decapping protein complex Dcp1a and Dcp2 in the P bodies is dependent on the presence of Edc4, as depletion of Edc4 blocks the accumulation of decapping enzymes in P bodies [17,18,19]. mTORC1 inhibition increases the expression of decapping proteins in yeast [20]; However, no direct evidence exists regarding involvement of mTORC1 in the regulation of Edc4.

In the present study we characterized Edc4 as an interacting partner of mTORC1. Moreover, the biological significance of Edc4 and mTORC1 interaction in the mRNA decapping process was investigated. Findings from the current study may help broaden our understanding of biological interplay of mTORC1 signaling in the mRNA decapping process.

## 2. Results

### 2.1. Edc4 Is an Interacting Partner of the Raptor Containing Component of mTORC1

**Figure 1:**
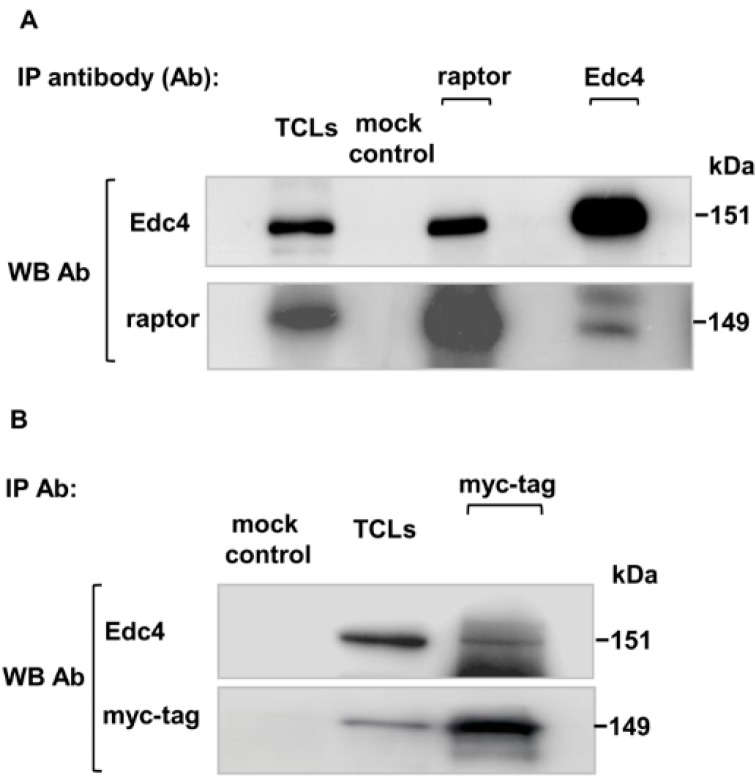
The Edc4 interaction with mTORC1: (**A**) CCRF-CEM cells were lysed, and Edc4 or raptor containing component of mTORC1 were co-immunoprecipitated using Edc4 and raptor antibody respectively. Immunoblotting with indicated antibodies confirmed co-precipitation of Edc4 with raptor and *vice versa*. No bands were detected in mock control (beads and whole cell lysates without adding antibody); and (**B**) CCRF-CEM cells were transiently transfected with myc-tag raptor pRK5 plasmid. After transfection, the cells were lysed and myc-tag raptor component of mTORC1 was specifically immunoprecipitated with myc-tag monoclonal antibody. The myc-tag IP elute was separated on SDS-PAGE and immunoblotted with corresponding antibodies which showed substantial association of Edc4 with the myc-tag raptor component (Western blot: WB, represents total cell lysates: TCLs).

In an attempt to identify novel interacting partners of mTORC1, we identified the Edc4 protein as a new mTORC1 interacting partner using nano-LC ESI Q-TOF MS/MS analysis [21]. These observations were subsequently confirmed by immunoblotting the raptor co-purified elute individually with raptor and Edc4 antibodies. A positive Edc4 signal was detected in the raptor specific immunoprecipitation (IP) elution, which identified Edc4 as a co-precipitating protein with the raptor component of mTORC1. Additionally, the reverse co-immunoprecipitation with Edc4 antibody produced a protein band corresponding to raptor which provides additional evidence for their interaction (Figure 1A).

To further verify the potential interaction of Edc4 with the raptor component of mTORC1, myc-tag raptor pRK5 plasmid was transiently transfected in CCRF-CEM cells. Myc-tag raptor component of mTORC1 was specifically immunopurified with monoclonal myc-tag antibody. The myc-IP and mock control elutes were separated on SDS-PAGE followed by immunoblotting. A strong myc-tag raptor signal was detected in myc-IP. This confirmed successful transfection of myc-tag raptor pRK5 plasmid and the immunoprecipitation of the myc-tag raptor component of mTORC1. Immunoblotting with Edc4 antibody revealed an Edc4 signal in the myc-IP elutes (Figure 1B). The presence of Edc4 in both the endogenous and exogenous purification of mTORC1 established Edc4 as a new interacting protein of mTORC1.

### 2.2. Edc4 Is an Interacting Partner of the Raptor Containing Component of mTORC1 but not mTORC2

**Figure 2:**
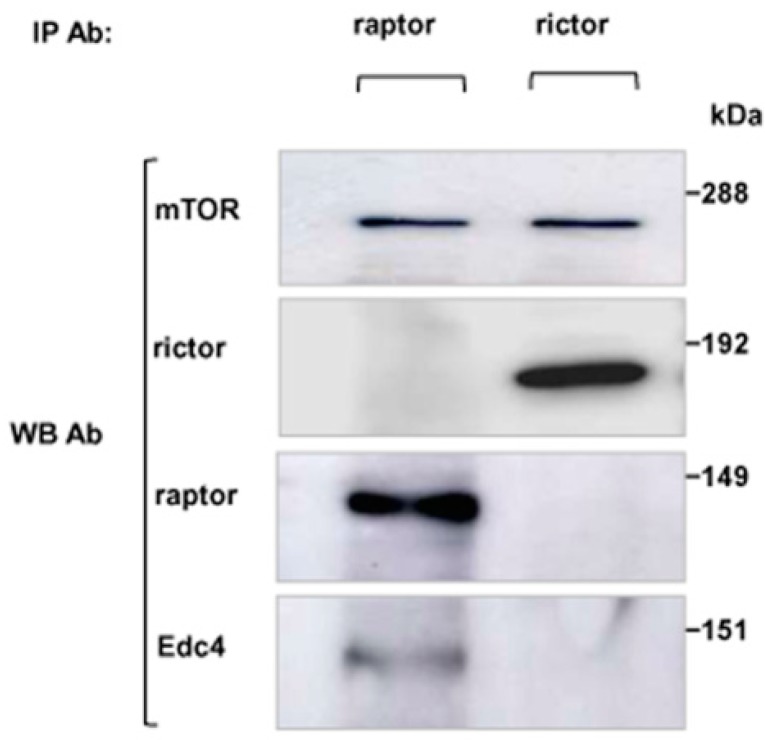
Edc4 interaction with mTORC1, but not with mTORC2: CCRF-CEM cells were lysed and endogenous mTOR complexes were immunopurified using raptor and rictor antibodies, respectively. The IP elutes were resolved on SDS-PAGE and immunoblotted with indicated antibodies which confirmed that Edc4 was only associated with mTORC1 but not with mTORC2.

It was investigated that whether Edc4 was associated with only raptor containing mTORC1 or whether it interacted with rictor containing mTORC2 as well. mTOR complexes from CCRF-CEM cell lysates were immunopurified with raptor and rictor antibodies. Raptor and rictor signals in the corresponding IP elutes were confirmed to check the specificity of our purifications. An mTOR signal was found in both raptor and rictor IPs which indicated co-immunoprecipitation of mTOR complexes. Immunoblotting with Edc4 specific antibody detected a band corresponding to Edc4 in the raptor IP elutes; However, no Edc4 signal was detected in the rictor IP elutions (Figure 2). This indicated that Edc4 was only associated with the raptor containing component of mTORC1 and that Edc4 did not interact with mTORC2.

### 2.3. Edc4 Co-Localized with Raptor Containing mTOR Complex

**Figure 3:**
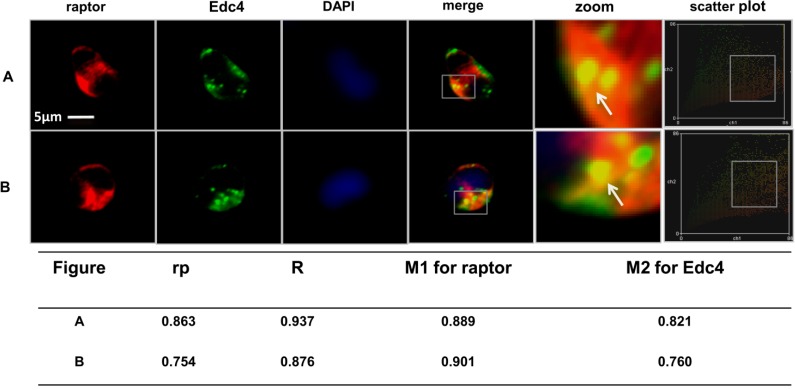
Edc4 co-localization with raptor component of mTORC1: CCRF-CEM cells were grown, fixed, permeabilized and both rabbit anti-Edc4 and mouse anti-raptor were added and incubated overnight. Fluorescent signals were detected using labeled anti-mouse Cydye3 and anti-rabbit Alexa Fluor 488. Raptor and Edc4 co-localization in CCRF-CEM cells was analyzed using an Axiovert 200M confocal microscope and processed with Image J software. The yellow overlay images demonstrate the high co-localization of raptor (red) with Edc4 (green) in processing bodies (P bodies, arrows indicate P bodies). The scatter plot of the individual pixels was obtained from two source images*.* Quantitative co-localization analysis illustrates increased co-localized pixels of Edc4 with raptor. At least 30 cells were observed per experiment (Scale bars = 5 μm) and experiments were repeated five times (only two experimental replicates **A** and **B** are shown). DAPI (4,6-diamidino-2-phenylindole) blue colour indicates cell nucleus.

Endogenous and myc-tag purification of mTORC1 and co-immunoprecipitation assays provided considerable evidence that Edc4 is an mTORC1 associated protein. Simultaneous immunofluorescence analysis of Edc4 (green) and raptor (red) was employed to view the co-localization of Edc4 with the raptor component of mTORC1. The merged yellow color reflects co-localization of Edc4 and raptor in P bodies (Edc4 is a marker of P bodies) [18] (Figure 3A,B). The images were processed to quantitatively determine the extent of co-localization using WCIFImage J (Wright Cell Image Facility, Toronto, ON, Canada). The graphical display as a scatter plot of raptor and Edc4 pixels appeared as yellow hues near the center of the *X* and *Y* axis. Statistical correlation using Pearson’s method [22] for two independent experiments showed coefficients of 0.863 and 0.754 respectively which suggested a high degree of co-occurrence of the raptor component of mTORC1 and Edc4. Similarly the Mander’s overlap coefficients (*R*) [23] were 0.937 and 0.876 respectively, indicating a high co-localization of pixels representing raptor and Edc4. Furthermore, the Mander’s co-localization coefficients for channel 1 (M1) were 0.889 and 0.901 from two consecutive experiments, describing the maximum number of raptor pixels co-localized with Edc4. The channel 2 co-localization coefficients (M2) were 0.821 and 0.760 respectively which represents the contribution of Edc4 channels co-localizing with raptor component of mTORC1 (Figure 3A,B).

### 2.4. Leucine Starvation and Rapamycin Treatment Enhanced Total Edc4 Protein Expression

**Figure 4:**
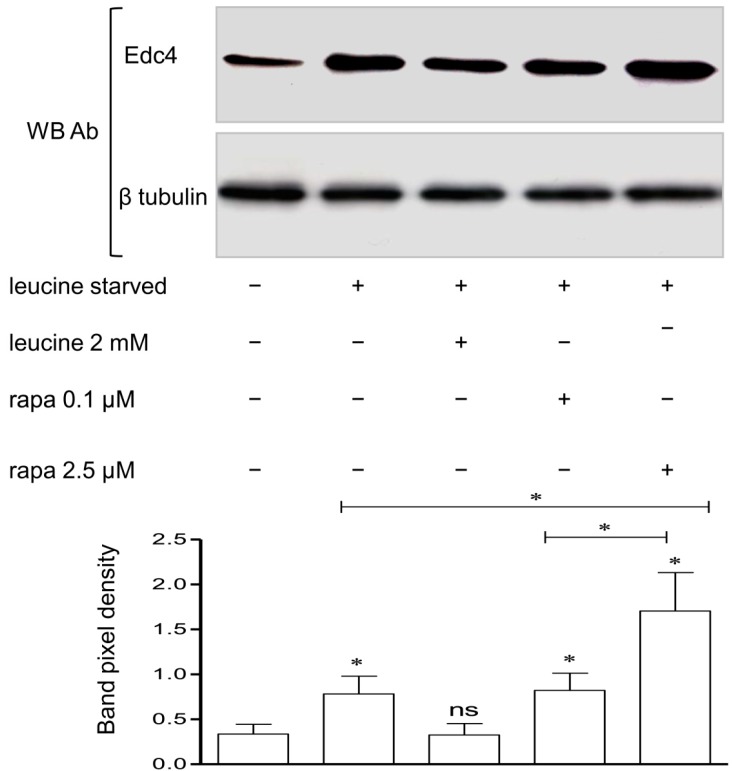
Leucine starvation and rapamycin treatment increased Edc4 protein expression: CCRF-CEM cells were grown, treated and lysed as described in methods section. Immunoblotting with Edc4 antibody detected significant change in the Edc4 expression in leucine starved and rapamycin treated cells as compared to control (without starvation). β tubulin was used as a loading control. The representative data present mean ± SEM of at least five independent experiments and the significance was determined by Student’s *t*-test (* *p* < 0.05; ns: Non-significant).

Leucine starvation and rapamycin treatment are both known to inhibit mTORC1 signaling [3]. In order to check for the influence of leucine or rapamycin on Edc4 expression, T cells were first leucine starved for two hours and then either stimulated for 30 min with leucine or treated for 1 h with rapamycin. Both leucine starvation and rapamycin treatment significantly increased the expression of Edc4 as demonstrated by immunoblotting in contrast to results with leucine stimulated cells or cells grown in complete (regular) medium (Figure 4).

### 2.5. Edc4 and Raptor Interaction Was Rapamycin Sensitive and Rapamycin Reduced the Amount of Total Serine Phosphorylated Edc4

**Figure 5:**
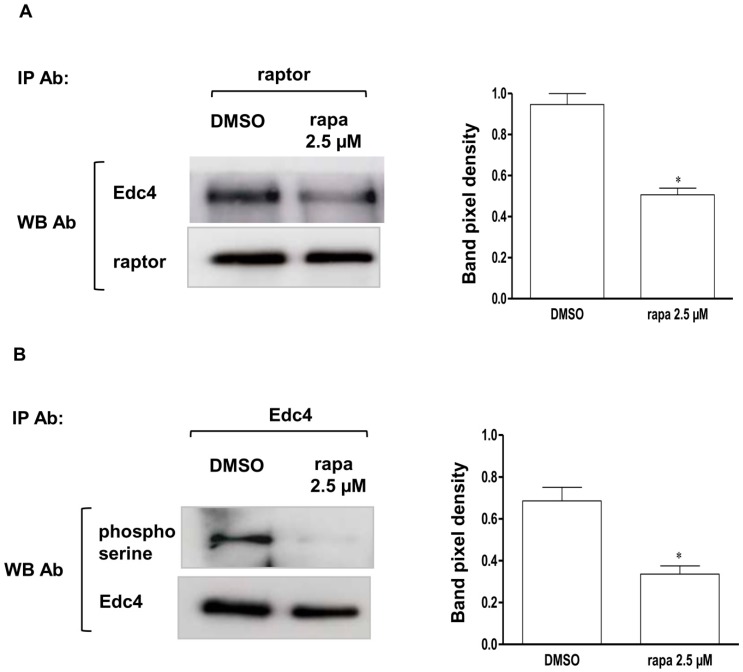
Edc4 and raptor interaction is rapamycin sensitive and rapamycin reduced Edc4 phosphorylation of serine residues: (**A**) CCRF-CEM cells were treated and lysed as described in the methods section. Endogenous mTORC1 was specifically immunoprecipitated using raptor antibody and resolved on the gel. Immunoblotting with corresponding antibodies detected decreased Edc4 signal in the treated as compared to non-treated controls (*n* = 4); and (**B**) CCRF-CEM cells were treated and lysed. Edc4 was specifically immunoprecipitated using Edc4 antibody and the IP elute was resolved on SDS-PAGE. Immunoblot analysis with phosphoserine antibody detected decreased phosphorylation signal in the rapamycin treated samples as compared to DMSO. The representative data are mean ± SEM of at least four independent experiments and the significance was determined by Student’s *t*-test (* *p* < 0.05).

In order to further explore the interaction between Edc4 and the raptor component of mTORC1, T cells were treated with rapamycin and DMSO (dimethylsulfoxid) for one hour. Cells were then lysed with CHAPS {(3-[(3-Cholamidopropyl)dimethylammonio]-1 propanesulfonate)} buffer followed by mTORC1 specific purification with raptor antibody. Immunoblotting showed almost equimolar amounts of raptor immuoprecipitation in rapamycin and DMSO treated samples; However, immunblotting with Edc4 antibody detected only a weak Edc4 signal in the rapamycin treated samples as compared to DMSO treated samples. This suggests that the Edc4 and raptor interaction was decreased by rapamycin inhibition of mTORC1 (Figure 5A). We further hypothesized that since mTOR is a serine threonine enzyme [24], it might regulate Edc4 via phosphorylation. To understand the involvement of mTORC1 in Edc4 regulation, cells were treated with rapamycin and DMSO followed by specific immunoprecipitation of Edc4. The Edc4 IP samples were immunoblotted with phosphoserine antibody. Decrease in the phosphorylated Edc4 serine was detected in the samples following rapamycin treatment (Figure 5B). These results provide the first evidence that mTORC1 regulation of Edc4 is through phosphorylation of serine sites on Edc4.

**Figure 6:**
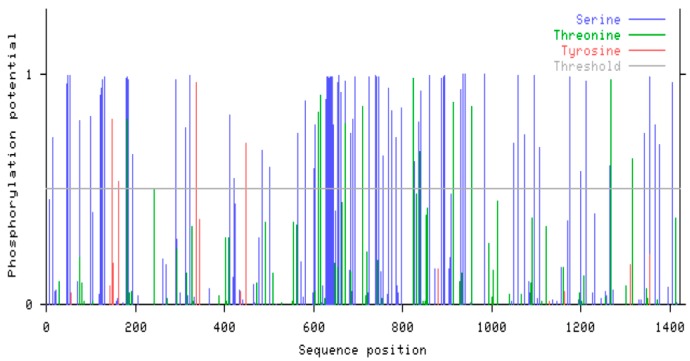
Predicted phosphorylation sites in Edc4 protein sequence: *In silico* Edc4 protein sequence analysis revealed Edc4 as a highly phosphorylated protein. A total of 86 serine, 11 threonine and 4 tyrosine phosphorylation sites were predicted in the Edc4 protein sequence where blue, green and red spectral lines represent serine, threonine and tyrosine respectively. There are 16 consecutive serine phosphorylation sites near the 600 amino acid sequence position of Edc4.

Moreover, *in silico* Edc4 protein sequence analysis (NetPhos 2.0 server [25]) revealed Edc4 as a serine rich protein (Figure 6).

### 2.6. Rapamycin Enhanced mRNA Decapping Activity

**Figure 7:**
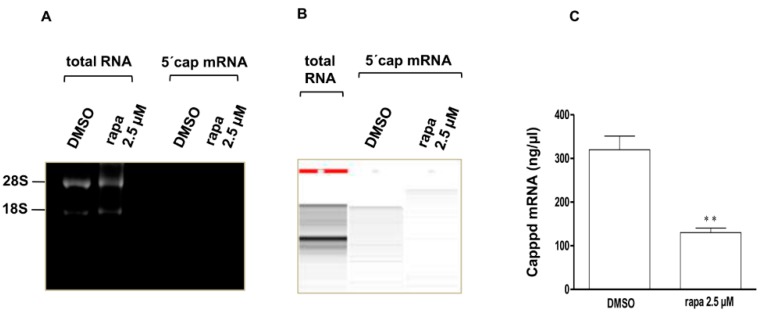
Rapamycin enhanced mRNA decapping activity: CCRF-CEM cells were treated with either rapamycin or vehicle control for one hour and (**A**) Total RNA was extracted from the cells, while the capped mRNAs were specifically isolated from total RNA using terminator exonuclease and lithium chloride precipitation. The removal of 18S and 28S rRNA from total 5'-capped mRNA was confirmed by 1.5% agarose gel electrophoresis; (**B**) After confirmation of 5'-capped mRNA purity, the total 5'-capped mRNA was run on the microchip gel and quantified; and (**C**) Bar diagram representation of five independent experiments (mean ± SEM), where significance was determined by Student’s *t*-test (** *p* < 0.005).

After observing that rapamycin induced a decrease in Edc4 interaction with raptor as well as in total serine phosphorylated Edc4, we hypothesized that mTORC1 inhibition may lead to increased mRNA decapping activity. To evaluate this, T cells were treated with rapamycin and 5'-capped mRNA was specifically isolated and quantified. A significant decrease in the total amount of 5'-capped mRNA was observed following rapamycin treatment as compared to control, suggesting that decapping activity was increased as a result of rapamycin induced mTORC1 inhibition (Figure 7).

## 3. Discussion

Edc4 is an important member of the mRNA decapping enzyme complex and has a suggested role in miRNA-mediated translational repression [14,16]. Edc4 is an essential constituent of P bodies and accelerates the mRNA decay process [17]. In human cells Edc4 exists as a multimeric protein having multiple WD40 (Trp-Asp) repeats at the *N*-terminus [17]. These repeats are known as the protein-protein interaction domain and serve as a scaffold for building protein complexes [26,27]. In some cases they play a role in recruiting phosphorylated proteins to the enzyme active sites [28]. The WD40 repeat domains of raptor and G β subunit-like (GβL), which are were previously identified as interacting proteins of mTORC1, are likely to play an important role in mTORC1 functions [3,29]. The existence of WD40 repeats in Edc4 might be involved in its interaction with mTORC1. The *C*-terminal region of Edc4 is conserved and is responsible for its localization in P bodies [18]. Rapamycin, an mTORC1 specific inhibitor, modulates mRNA turnover by increasing the expression of decapping protein in *S. cerevisiae*. This reflects the involvement of mTOR signaling in mRNA degradation [20]. In the present study, Edc4 was identified in the mTORC1 specific endogenous purification as well as in the myc-tag pulldown of mTORC1. The Edc4 signal was only detected in mTORC1 purifications while it was absent from the mTORC2 specific purifications. Therefore, our experimental evidence suggests that Edc4 is associated with mTORC1 and might not interact with the mTORC2 loop of the mTOR signaling pathway.

We further found that the raptor component of mTORC1 co-localized with Edc4 in both the cytoplasm and in the cytosolic P bodies. In P bodies, mRNAs are either degraded or stored for return to translation [8]. Edc4 is the key component of P bodies and is even used as a marker to localize P bodies [17,18]. The cap binding protein eIF4E and the 4E-transporter (4E-T, a negative regulator of eIF4E), co-localized with the P bodies [30]. eIF4E is a potential target for 4E-BP1 inhibitory action [31]. mTORC1 phosphorylates 4E-BP1, and prevents 4E-BP1 binding to eIF4E which allows eIF4E to take part in the translation initiation process [32]. We hypothesize that the presence of mTORC1 within the P bodies might allow for the transition of a stored mRNA to a translationally competent state or regulate mRNA decapping by interacting with Edc4 in the P bodies. This possibility needs further investigation. Quantitative co-localization fluorescent labeled antibody studies of Edc4 with the raptor component of mTORC1 revealed a significant extent of co-occurrence between two different antibody bound fluorescent labels with separate emission spectra. This suggests that the co-localized proteins are in very close proximity or it might even reside at the same physical location [22]. Co-localization of raptor (red pixels) and Edc4 (green pixels) in the scatter plots exhibited high co-localization between the raptor component of mTORC1 and Edc4.

Furthermore, Pearson’s correlation coefficients (*rp*) were used to measure the extent of co-occurrence between two fluorescence channels. Pearson’s coefficients range from −1 to 1, with a value of −1 indicating a total lack of overlap between pixels from the two images, and a value of 1 representing perfect correlation [22]. The Pearson’s correlation coefficients demonstrated a high co-localization between Edc4 and raptor counterpart of mTORC1. Mander’s overlap coefficient (*R*) which is insensitive to fluorochrome concentration fluctuations and photobleaching was calculated [22]. This coefficient ranges between 0 and 1, with 1 being highly co-localized pixels and zero being the least co-localized pixels [23]. The calculated R coefficients for channel 1 (M1) and channel 2 (M2) both confirmed a high degree of overlapping pixels between Edc4 and raptor protein in P bodies.

In order to elucidate the mechanism involved in the Edc4 and mTORC1 interaction, we used leucine, a known stimulator of mTORC1 mediated translation [3,33]. Nutrient starvation inhibits mTOR signaling and causes an increased turnover of a subset of mRNA in yeast [20]. Leucine starvation increased mRNA and protein expression of transcription factors [34]. In our experiments, leucine starvation induced Edc4 expression as compared to the leucine stimulated and complete medium supplemented cells. This indicates that regulation of mTORC1 kinase activity by leucine [3] increased Edc4 expression.

To gain further insight into the mTORC1 involvement in the regulation of Edc4, rapamycin, a specific inhibitor of mTORC1 was employed. Rapamycin treatment, which should mimic nutrient starved conditions, also modulated Edc4 expression providing convincing evidence of mTORC1 involvement in the regulation of Edc4. These observations are also in the line with previous studies where rapamycin was reported to increase the expression of decapping proteins and mRNA turnover [20,35]. We further demonstrated that the mTORC1 inhibition induced by rapamycin decreased mTORC1 interactions with Edc4. One possible explanation is that interaction of the raptor component of mTORC1 with Edc4 might be responsible for control of Edc4 activity in the mRNA decapping process. This mTORC1 interaction was decreased by rapamycin treatment and thus more Edc4 was available to take part in the mRNA decapping process.

**Figure 8:**
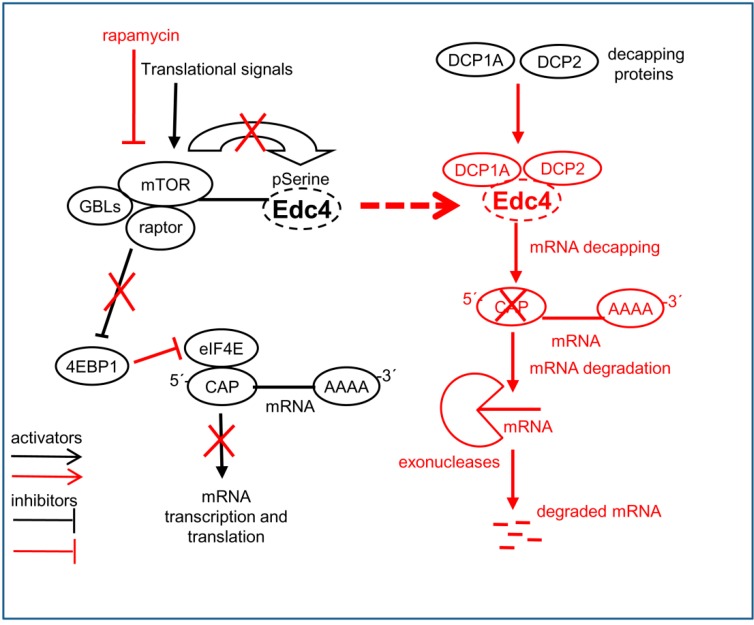
mTORC1 interplay in mRNA decapping through interaction with Edc4: The schematic diagram (Edc4 box) shows that mTORC1 interacted with Edc4 to keep its expression at the basal level by inhibiting Edc4 through serine phosphorylation (white bold arrow). This hyper-phosphorylated Edc4 was then no longer available for mRNA decapping activity in the mRNA decay process. Treatment of cells with rapamycin led to the inhibition of mTORC1 kinase activity, resulting in Edc4 dephosphorylation. This dephosphorylated Edc4 probably leads to activation of the decapping machinery and increased mRNA degradation.

The Edc4 is a phospho-protein with 86 serine, 11 threonine and 4 tyrosine predicted phosphorylation sites (NetPhos 2.0 server [25]). In total 29 phosphorylation sites including 19 phosphoserine, 3 phosphotyrosine and 7 phosphothreonine of yet unknown functional significance have been confirmed by mass spectrometric analysis (Phosphosite server [36]). The mTOR is a well-characterized serine threonine kinase complex while Edc4 is a serine rich protein which has a stretch of 16 consecutive serine rich residues [17]. Thus, Edc4 could be a target for various kinases including mTOR. To establish the involvement of mTORC1 in Edc4 regulation, we examined the effect of rapamycin treatment on the phosphorylation status Edc4. A significant decrease was observed in total serine phosphorylated Edc4 protein signal after rapamycin treatment which indicated a role of mTORC1 kinase in Edc4 regulation. While investigating whether rapamycin had any effect on the total amount of 5'-capped mRNA in cells, we observed a substantial decrease in the amount of 5'-capped mRNA associated with rapamycin treatment. We cannot exclude the possibility that the decrease in capped mRNA was due to rapamycin mediated inhibition of elF4E; However, the increase in the decapping protein expression following rapamycin treatment [20] is in line with our hypothesis. These findings strongly suggest a regulatory role for mTORC1 in the total amount of 5'-capped mRNA in cells as a result of decreased cellular mRNA decapping activity. Based on these results, we speculate that on receiving translation signals, mTOR kinase activity is increased. This on one hand increases mRNA transcription and translation, while on the other hand it may simultaneously also phosphorylate Edc4 and keep its expression checked. In the phosphorylated form, Edc4 may be bound to mTORC1, and not available as a scaffolding protein for DCP1 and DCP2. Consequently this would keep the decapping machinery incomplete, which could prolong mRNA half-life and increase its availability for translation. However, agents causing negative signals like rapamycin which inhibits the mTOR kinase activity, could lead to dephosphorylation and release of Edc4 from mTORC1 binding. Released Edc4 could complex with DCP1 and DCP2, complete the decapping machinery, and thereby enhance mRNA degradation (Figure 8).

## 4. Experimental Section

### 4.1. Antibodies and Reagents

Antibodies and reagents were obtained from the following sources: myc-tag and mTOR antibodies from Cell Signaling Technology (Danvers, MA, USA). Raptor anti-mouse and anti-rabbit antibodies from Millipore (Schwalbach, Germany). Rictor antibody from Bethyl Laboratories (Boston, TX, USA) and β-tubulin antibody was from BioVendor (Heidelberg, Germany). Edc4 antibody from Abcam (Cambridge, UK). HRP-labelled anti-mouse, anti-goat, and anti-rabbit secondary antibodies were from Bio-Rad (Munich, Germany).

Anti-phosphoserine, anti-rabbit Alexa Fluor 488 and anti-mouse Cydye3 labelled antibodies, dynabeads G, TRIzol, lipofectamine LTX, plus reagent, Opti MEM, were from Invitrogen (Darmstadt, Germany). Triton X-100 lysis buffer was from Cell Signaling Technology. CHAPS buffer was from Applichem (Darmstadt, Germany). Rapamycin was from LC Laboratories (Woburn, MA, USA). Complete protease and phosphatase inhibitors cocktail were from Roche (Mannheim, Germany). Leucine, and RPMI-1640 without leucine were from Sigma-Aldrich (Steinheim, Germany). RPMI-1640, DMEM, phosphate buffer saline (PBS), penicillin and streptomycin were from PAA Laboratories (Colbe, Germany). Capped mRNA isolation kit was from Epicenter Biotechnologies (Madison, WI, USA). Myc-tag Co-immunoprecipitation (Co-IP) kit was from Thermo Scientific Pierce (Rockford, IL, USA). Myc-tag raptor pRK5 plasmid was provided by Doss Sarbassove (The University of Texas, Austin, TX, USA).

### 4.2. Cell Culture

Human T lymphoblasts (CCRF-CEM) cells were purchased from DSMZ (German collection of microorganisms and cell cultures (Braunschweig, Germany)). Cells were grown in RPMI-1640 medium supplemented with 10% FCS, 100,000 U/L penicillin and 100 µg/L streptomycin (Biochrome, Berlin, Germany) under 95% humidity and 5% CO_2_ conditions at 37 °C. Cell confluency was regularly checked, and the medium was changed accordingly.

### 4.3. Cell Lysis and Reverse Co-Immunoprecipitation (Co-IP)

CCRF-CEM Cells (60 million) were rinsed with cold PBS and lysed on ice cold 0.3% CHAPS buffer to isolate mTOR complexes [37]. Reverse Co-IPs were performed as previously described [21]. Briefly cell debris was removed from the cell lysate by centrifugation. Antibodies for Co-IP were added to the lysate and incubated at 4 °C. Dynabeads G were added to the antibody and lysate mixture, and incubated for overnight at 4 °C. Mock control (beads and whole cell lysates without adding antibody) was incorporated to exclude false interaction of lysate proteins with the dynabeads G. Immunoprecipitates and mock controls were washed with 0.3% CHAPS buffer. Samples were eluted in 2× Laemmli buffer, and then resolved on SDS-PAGE. For experiments with cell lysates, Triton X-100 containing lysis buffer was used.

### 4.4. Mammalian Cells Transfection and Myc-Tag mTORC1 Purification

CCRF-CEM cells were transfected with myc-tag raptor pRK5 plasmid [21]. Lipofectamine LTX and Plus reagents were used as per vendor recommendations (Invitrogen, Darmstadt, Germany). The mixture was added to the cells and incubated at 37 °C for 48 h. Transfected cells were lysed on ice cold 0.3% CHAPS buffer to isolate mTOR containing complexes as described earlier [37]. Cell lysates were separated from insoluble cell debris by centrifugation. A myc-tag Co-IP kit (Thermo Scientific Pierce, Rockford, IL, USA) was used to pull down myc-tag raptor component of mTORC1. Briefly, lysates and myc-tag monoclonal antibody conjugated beads were incubated overnight at 4 °C. Mock controls were run as a negative control. Immunoprecipitates were washed with CHAPS buffer, and then eluted.

### 4.5. SDS-PAGE and Immunoblot Analysis of Co-IP Samples

Immunoprecipitated elutes were resolved on SDS-PAGE and blotted onto PVDF membrane (Millipore, Schwalbach, Germany) using the semidry Trans-Blot Semi Dry cell system (Bio-Rad, Munich, Germany) as described previously [21]. Briefly, the membrane was blocked with skimmed milk powder prepared in TBS-T buffer (50 mmol/L Tris-HCl (pH 7.5), 0.05% Tween 20, 200 mmol/L NaCl) and then washed with TBS-T buffer. Primary antibody was added for overnight incubation at 4 °C. After washes with TBS-T, the membrane was incubated in HRP-conjugated secondary antibodies and then washed in TBS-T. Enhanced chemiluminescent (ECL) reagent (GE Healthcare, Buckinghamshire, UK) was used to detect signals on the blot and then developed on Amersham Hyperfilm (GE Healthcare). Signal quantification for each immunoblot was done using the Lab Image software version 2.71 (Kapelan, Leipzig, Germany).

### 4.6. Confocal Immunofluorescence Microscopy

CCRF-CEM cells grown on 8 chamber well slides (Lab-Tek™ II (Thermo Scientific Pierce) were fixed in freshly prepared 3.7% paraformaldehyde for 5 min at room temperature. The cells were first rinsed and permeated with 0.2% Triton X-100 in PBS for 15 min and then incubated with 1% BSA in PBS for 30 min to block nonspecific binding of antibodies. After thorough rinsing in PBS, rabbit anti-Edc4 (1:300) and mouse anti-raptor (1:300) antibodies were added to the cells, and the mixture incubated overnight at 4 °C. After washing, the cells were probed with fluorescein labeled secondary antibodies, anti-mouse Cydye3 (1:200) and anti-rabbit Alexa Fluor 488 (1:200) for one hour at room temperature. Nuclei were counter-stained with 4,6-diamidino-2-phenylindole (DAPI) for 10 min, mounted with Fluoromount (DAKO, Hamburg, Germany), and then visualized with a confocal microscope (Axiovert 200M, Carl Zeiss, Jena, Germany). The DAPI staining in the blue channel has been shown to indicate the outline of the nuclei [38]. The three channel images and an overlay image of red and green channels were recorded using the Axiovision software (Carl Zeiss, Jena, Germany). Quantitative co-localization analysis was carried out using the WCIF Image J software (http://www.uhnres.utoronto.ca/facilities/wcif/imagej).

### 4.7. Leucine and Rapamycin Treatments

Cells were grown for 24 h in RPMI-1640 supplemented with 10% fetal calf serum (FCS). The medium was then replaced with RPMI-1640 without leucine for 2 h and then stimulated with 2 mM leucine for 30 min [39] or treated with either 0.1 or 2.5 µM rapamycin (the rapamycin concentration at which approximately 70% of CCRF-CEM cell proliferation inhibition occurs [40]) for one hour. Cells were then lysed, their contents were resolved on SDS-PAGE and immunoblotted to observe changes in the expression of Edc4 after both leucine and rapamycin treatment.

### 4.8. RNA Isolation

Total cellular RNA was isolated using the TRIzol method [41]. Briefly, CCRF-CEM cells were grown, and treated with 2.5 µM rapamycin, or vehicle control (DMSO) for one hour followed by homogenization in TRIzol reagent. RNA was extracted using a chloroform/isopropanol precipitation method. The RNA concentration was quantified with an Agilent 2100 Bioanalyzer (Agilent Technologies, Waldbronn, Germany). The integrity of the extracted total RNA and capped mRNA was ascertained by electrophoresis on 1.5% agarose gels.

### 4.9. Capped mRNAs Isolation and Quantification

Capped mRNAs were isolated from total RNA as described by the vendor (Epicenter Biotechnologies, Madison, WI, USA). Briefly, 5 µg total RNA was incubated with the reaction mixture (RNase-free water, 10x reaction buffer A, riboGuard RNase inhibitor, and 1 unit of terminator exonuclease) at 30 °C for 60 min in a thermocycler. The reaction was terminated by adding stop solution (EDTA 5 mM). Lithium chloride precipitation was performed at −20 °C for 30 min (to enrich mRNA and to get rid of EDTA, tRNA, and other small RNA species), followed by centrifugation at 14,000 rpm for 30 min at 4 °C. The mRNA pellet was then washed with 70% ethanol to remove residual salts. The RNA pellet was resuspended in RNase-free water. The successful removal of 18S and 28S rRNA from total RNA content was confirmed by 1.5% agarose gel electrophoresis. Capped mRNAs were quantified with use of an Agilent 2100 Bioanalyzer (Agilent technologies, Waldbronn, Germany).

### 4.10. Statistical Analysis

All experiments in this study were repeated at least four times and results are expressed as mean ± SEM with significance measured using the Student’s *t*-test (*p* < 0.05).

## 5. Conclusions

In the present study, we characterized Edc4 as mTORC1 interacting protein. mTORC1 inhibition by rapamycin, and co-localization analysis provided additional evidence for Edc4 and mTORC1 interactions. Modulation of Edc4 expression and mRNA decapping after rapamycin treatment suggests mTORC1 involvement in Edc4 regulation. A decrease in the phosphorylation of Edc4 after mTORC1 inhibition suggests a role for mTORC1 in the decapping process. These findings highlight the potential role of mTORC1 in mRNA decapping via its interaction with Edc4. Further studies are required to provide a more complete understanding of the biological interplay between mTORC1 signaling and the mRNA decapping process.

## References

[1] Zhou H., Huang S. (2010). mTOR signaling in cancer cell motility and tumor metastasis. Crit. Rev. Eukaryot. Gene Expr..

[2] Laplante M., Sabatini D.M. (2012). mTOR signaling in growth control and disease. Cell.

[3] Kim D.H., Sarbassov D.D., Ali S.M., King J.E., Latek RR., Erdjument-Bromage H., Tempst P., Sabatini D.M. (2002). mTOR interacts with raptor to form a nutrient-sensitive complex that signals to the cell growth machinery. Cell.

[4] Sarbassov D.D., Ali S.M., Kim D.H., Guertin D.A., Latek R.R., Erdjument-Bromage H., Tempst F., Sabatini D.M. (2004). Rictor, a novel binding partner of mTOR, defines a rapamycin-insensitive and raptor-independent pathway that regulates the cytoskeleton. Curr. Biol..

[5] Caron E., Ghosh S., Matsuoka Y., Ashton-Beaucage D., Therrien M., Lemieux S., Perreault C., Roux P.P., Kitano H. (2010). A comprehensive map of the mTOR signaling network. Mol. Syst. Biol..

[6] Laplante M., Sabatini D.M. (2009). mTOR signaling at a glance. J. Cell Sci..

[7] Coller J., Parker R. (2005). General translational repression by activators of mRNA decapping. Cell.

[8] Parker R., Sheth U. (2007). P bodies and the control of mRNA translation and degradation. Mol. Cell.

[9] Wang X., Proud C.G. (2011). mTORC1 signaling: What we still do notdo not know. J. Mol. Cell Biol..

[10] Chao S.K., Horwitz S.B., McDaid H.M. (2011). Insights into 4E-BP1 and p53 mediated regulation of accelerated cell senescence. Oncotarget.

[11] Eulalio A., Behm-Ansmant I., Izaurralde E. (2007). P bodies: At the crossroads of post-transcriptional pathways. Nat. Rev. Mol. Cell Biol..

[12] Huang L., Mollet S., Souquere S., Le R.F., Ernoult-Lange M., Pierron G., Dautry F., Weil D. (2011). Mitochondria associate with P-bodies and modulate microRNA-mediated RNA interference. J. Biol. Chem..

[13] Ernoult-Lange M., Benard M., Kress M., Weil D. (2012). P-bodies and mitochondria: Which place in RNA interference?. Biochimie.

[14] Fenger-Gron M., Fillman C., Norrild B., Lykke-Andersen J. (2005). Multiple processing body factors and the ARE binding protein TTP activate mRNA decapping. Mol. Cell.

[15] Bloch D.B., Rabkina D., Quertermous T., Bloch K.D. (1994). The immunoreactive region in a novel autoantigen contains a nuclear localization sequence. Clin. Immunol. Immunopathol..

[16] Brodersen P., Sakvarelidze-Achard L., Bruun-Rasmussen M., Dunoyer P., Yamamoto Y.Y., Sieburth L., Voinnet O. (2008). Widespread translational inhibition by plant miRNAs and siRNAs. Science.

[17] Yu J.H., Yang W.H., Gulick T., Bloch K.D., Bloch D.B. (2005). Ge-1 is a central component of the mammalian cytoplasmic mRNA processing body. RNA.

[18] Jinek M., Eulalio A., Lingel A., Helms S., Conti E., Izaurralde E. (2008). The *C*-terminal region of Ge-1 presents conserved structural features required for P-body localization. RNA.

[19] Fan S.J., Marchand V., Ephrussi A. (2011). Drosophila Ge-1 promotes P body formation and oskar mRNA localization. PLoS One.

[20] Albig A.R., Decker C.J. (2001). The target of rapamycin signaling pathway regulates mRNA turnover in the yeast *Saccharomyces cerevisiae*. Mol. Biol. Cell.

[21] Rahman H., Qasim M., Oellerich M., Asif A.R. (2014). Identification of the novel interacting partners of the mammalian target of rapamycin complex 1 in human CCRF-CEM and HEK293 cells. Int. J. Mol. Sci..

[22] Adler J., Parmryd I. (2010). Quantifying colocalization by correlation: The Pearson correlation coefficient is superior to the Mander’s overlap coefficient. Cytom. A.

[23] Manders E.M.M., Verbeek F.J., Aten J.A. (1993). Measurement of co-localization of objects in dualcolor confocal images. J. Microsc..

[24] Yang Q., Guan K.L. (2007). Expanding mTOR signaling. Cell Res..

[25] Blom N., Gammeltoft S., Brunak S. (1999). Sequence and structure-based prediction of eukaryotic protein phosphorylation sites. J. Mol. Biol..

[26] Smith T.F., Gaitatzes C., Saxena K., Neer E.J. (1999). The WD repeat: A common architecture for diverse functions. Trends Biochem. Sci..

[27] Gudkova D., Panasyuk G., Nemazanyy I., Zhyvoloup A., Monteil P., Filonenko V., Gout I. (2012). EDC4 interacts with and regulates the dephospho-CoA kinase activity of CoA synthase. FEBS Lett..

[28] Yaffe M.B., Elia A.E. (2001). Phosphoserine/threonine-binding domains. Curr. Opin. Cell Biol..

[29] Kim D.H., Sarbassov D.D., Ali S.M., Latek R.R., Guntur K.V., Erdjument-Bromage H. (2003). GβL, a positive regulator of the rapamycin-sensitive pathway required for the nutrient-sensitive interaction between raptor and mTOR. Mol. Cell.

[30] Sheth U., Parker R. (2003). Decapping and decay of messenger RNA occur in cytoplasmic processing bodies. Science.

[31] Hay N., Sonenberg N. (2004). Upstream and downstream of mTOR. Genes Dev..

[32] Guertin D.A., Sabatini D.M. (2007). Defining the role of mTOR in cancer. Cancer Cell.

[33] Kim J., Guan K.L. (2011). Amino acid signaling in TOR activation. Annu. Rev. Biochem..

[34] Bruhat A., Jousse C., Carraro V., Reimold A.M., Ferrara M., Fafournoux P. (2000). Amino acids control mammalian gene transcription: Activating transcription factor 2 is essential for the amino acid responsiveness of the CHOP promoter. Mol. Cell. Biol..

[35] Ikari A., Sanada A., Sawada H., Okude C., Tonegawa C., Sugatani J. (2011). Decrease in transient receptor potential melastatin 6 mRNA stability caused by rapamycin in renal tubular epithelial cells. Biochim. Biophys. Acta.

[36] Hornbeck P.V., Chabra I., Kornhauser J.M., Skrzypek E., Zhang B. (2004). PhosphoSite: A bioinformatics resource dedicated to physiological protein phosphorylation. Proteomics.

[37] Peterson T.R., Laplante M., Thoreen C.C., Sancak Y., Kang S.A., Kuehl W.M. (2009). DEPTOR is an mTOR inhibitor frequently overexpressed in multiple myeloma cells and required for their survival. Cell.

[38] Trotta E., D’Ambrosio E., Ravagnan G., Paci M. (1995). Evidence for DAPI intercalation in CG sites of DNA oligomer [d(CGACGTCG)]_2_: A 1H NMR study. Nucleic Acids Res..

[39] Du M., Shen Q.W., Zhu M.J., Ford S.P. (2007). Leucine stimulates mammalian target of rapamycin signaling in C_2_C_12_ myoblasts in part through inhibition of adenosine monophosphate-activated protein kinase. J. Anim. Sci..

[40] Schultze F.C., Petrova D.T., Oellerich M., Armstrong V.W., Asif A.R. (2010). Differential proteome and phosphoproteome signatures in human T-lymphoblast cells induced by sirolimus. Cell Prolif..

[41] Chomczynski P. (1993). A reagent for the single-step simultaneous isolation of RNA, DNA and proteins from cell and tissue samples. Biotechniques.

